# Diet and Culture Among Chinese Patients Undergoing Hemodialysis: A Qualitative Study

**DOI:** 10.3389/fnut.2022.876179

**Published:** 2022-04-25

**Authors:** Yan Song, Jing Wang, Huan Liu, Xiaolan Chen, Minqi Zhan

**Affiliations:** ^1^Department of Fundamentals of Nursing, Medical School, Nantong University, Nantong, China; ^2^Department of Medical Nursing, School of Nursing, Fudan University, Shanghai, China; ^3^Department of Nephrology, Affiliated Hospital of Nantong University, Nantong, China

**Keywords:** hemodialysis, culture, diet, nutrition, qualitative research

## Abstract

A growing body of research showed that diet management, such as promoting protein and vitamin intake and food restriction play a crucial role in extending time to morbidity and mortality in patients undergoing hemodialysis. However, the current dietary recommendations in nutrition guidelines lack examination of cultural factors. The study aimed to understand the diet influenced by culture in Chinese patients undergoing hemodialysis. Semi-structured interviews were conducted for 23 patients, aged 23–75 years, undergoing hemodialysis in a local tertiary hospital. Interview questions mainly focused on patients' real experience about diet, and their perceptions and attitudes toward diet. Each interview was digitally recorded, and conventional content analysis was used to attain information. The majority of patients reserved Chinese traditional dietary habits about salt and calcium intake. Although Chinese herbal medicine was not consumed, dietary therapy including brown sugar and dates was included in the participants' diet. Eggs, broth, and seafood were three prominent preferences and taboos profoundly impacted by culture. Additionally, Chinese social culture influenced patients' dietary behavior of eating at home and knowledge attainment. Diet in Chinese patients undergoing hemodialysis was still strongly influenced by culture. Culturally sensitive interventions regarding the improvement of diet intake are urgently needed.

## Background

Hemodialysis (HD) is the most widely available system of life maintenance for patients with end-stage kidney diseases and is reported to be used by 87% of all the patients undergoing dialysis (1–3). Nowadays, patients needing dialysis is estimated between 4.902 and 7.083 million worldwide (4). The prevalence of patients undergoing HD in China has been increasing remarkably in recent years. The number was only 3,85,000 in 2015 (5) and was close to 5,00,000 (6) in 2017. The high prevalence of HD in China places an increasing burden on both families and society due to the significant increases in the incidences of obesity and diabetes in the foreseeable future (7).

Malnutrition and abnormal serum nutrient concentrations that are strongly associated with diet have been commonly seen in patients undergoing HD with a high risk of increased morbidity and mortality (8, 9). In 2020, the Kidney Disease Outcomes Quality Initiative (KDOQI) proposed an updated global clinical practice guideline for nutrition in chronic kidney disease, which included some dietary recommendations regarding nutritional intake including protein, trace elements and minerals for patients undergoing HD (10). Protein intake of 1.0–1.2 g/kg body weight per day is recommended to maintain a stable nutritional status in the KDOQI guide. However, daily protein intake (DPI) in most Chinese patients undergoing HD who also follow the guidelines was < 1.0 g/kg (11).

Although serum trace elements or minerals concentrations are only partly influenced by dietary intake in patients undertaking HD, it is required to alert Chinese patients' dietary habits and behaviors when facing undesirable serum concentrations compared to their counterparts in other countries. 54.5% of Chinese patients undergoing HD had serum phosphate levels > the normal range (1.7 mmol/L) (5). They had worse control of serum phosphate than their British counterparts, whose hyperphosphatemia accounted for 35.9% of the cohort (12). Higher calcium intake has previously been reported in European countries compared to the general population in China, which likely reflected different dairy intake between these countries (13). Dietary patterns may cause the higher proportion of patients with hypocalcemia (36.0%) in the Chinese patients undergoing HD (5), compared with the one in British patients (10.6%) who had calcium levels < 2.2 mmol/L (12).

Traditional Chinese Medicine with a history of thousand of years comprises of Yin and Yang theory, which represents two opposing and complementary forces and regulates the harmony between health and disease (14, 15). Food can also be categorized into Yin and Yang. Hence, the Chinese population generally believes that the preferences or taboos of food are of paramount importance to people's overall health and well-being, which influences the balance between Yin and Yang. The theory of dietary herbal medicines and dietary therapy are two crucial parts of the traditional Chinese medicine system as well. The pharmacological effects of dietary herbal medicines have been widely used to prevent or treat various diseases in China. Goji berry (*Lycium barbarum)* has always been described to work well in nourishing kidneys (16). Nowadays increasing studies showed that Lycium extracts, such as Europaeum and *Lycium barbarum* polysaccharides had the potential kidney protection (17, 18). Food is a diet providing necessary substances. In Chinese traditional culture, however, food is also regarded as medicine. Food therapy is the method of promoting health by using or adjusting food (19), which has been acknowledged by the Chinese population for more than 3,000 years (20). Additionally, Chinese people have always held a belief that animal organs correspond to human organs. If there is kidney injury, for example, then a pig's kidney can be eaten to strengthen the kidney function. Majority of Chinese health care professionals are often unaware of the influences of culture on diet management in patients undergoing HD as they are trained with western medical educational systems (14). Furthermore, Chinese patients undergoing HD generally follow the international consensus treatment rather traditional Chinese medicine. Therefore, establishing and implementing a culturally sensitive nutrition intervention requires a thorough understanding of the relationship between culture and diet in patients undergoing HD. This study aimed to understand the diet influenced by culture in Chinese patients undergoing hemodialysis.

## Method

The qualitative study was conducted in a tertiary hospital in the east of China. The details of the study were explained to the potential participants, and then written consent was obtained from all the participants who were willing to participate in the study. Ethical approval was given by the Ethics Committee of the Affiliated Hospital of Nantong University (Ref.2015-12). The research team includes two doctoral researchers in charge of designing, implementing and analyzing the study, two graduate students assisting the participants' recruitment and one nephrology consultant providing guidance.

### Participants Recruitment

Adult patients undergoing maintenance hemodialysis who participated in a larger quantitative study at a local tertiary hospital and expressed their willingness to participate in future studies were recruited. The exclusion criteria included age <18 years, receiving nutrition therapy, such as enteral nutrition or specific dietary restrictions, insufficient command over Mandarin or the dialect to give informed consent, or comply with the interview, and presence of speaking, hearing, and cognitive impairments. Maximum variation sampling (21) was adopted to increase the diversity of personal experiences within the given population (22). The participants were selected considering the wide range of demographics that are potentially associated with dietary intake or nutritional knowledge, including age, gender, residence, body mass index (BMI), education level, income, medical insurance, and living with family members.We explained the details of the consent form to patients who were willing to participate in the study and resolved their questions regarding the study. Recruitment was conducted until data saturation was achieved. Data saturation criteria referred to data replication and redundancy when additional interviews failed to uncover new ideas related to the study purpose (23).

### Data Collection

A semi-structured interview pattern was applied to participants that comprised open-ended questions and prompt. The initial interview questions were developed by two first authors, based on the literature review of studies focusing on exploring the perceptions and attitudes to diet intake and the real experience about diet management among patients undergoing hemodialysis (24–26). Questions were then revised and added based on consultation with one health care professional. Two participants were randomly selected for a pilot interview, and the final interview guide was formed according to the interview results. All the interview questions were initially developed in Mandarin. Additionally, questions related to dietary culture were not initially included in the interview guide. However, researcher would make a detailed inquiry when patients spontaneously mentioned the topic. The interview guide included, “What do you think about protein for patient undergoing HD, and how do you have protein?”, “What is your understanding of diet restriction in HD?”, “Could you please name the foods that should be restricted as much as possible and tell me why you think it should be prohibited for patients?”, “Will you purposely choose any food?” and “How do you gain your dietary knowledge?” Some of the questions were slightly modified based on the participants' replies. For example, few participants perceived sodium control or calcium intake as a vital part of dietary management, and the researcher required them to express their views on sodium or calcium management. Furthermore, the researchers added a series of questions for successive interviews based on the content analysis results of previous interviews. These included, “How do you maintain your hemoglobin level in a normal range?”, “Have you ever tried Chinese herbal medicine and what do you think of it?”, and “Would you like to actively consult your doctors or nurses for dietary guidance?” Every interview was conducted with one participant and the first author in a meeting room in the hospital with the sign of “Interview in process; do not disturb” on the door. The interview times were chosen by patients as per their preference. Generally, it took participants 40 to 60 min for every interview, depending on the amount of information they wanted to share. All the interviews were digitally recorded and the transcripts were uploaded to NVivo 11.

### Data Analysis

The two first authors independently read each transcript several times to gain insight into the individual participant's perception about diet by applying conventional content analyses (27). In the first cycle of coding, meaningful or recurring views of patients in the transcripts were coded to identify key points. The two primary authors used notes to keep a track of the rationale for coding. Their notes were double-checked with the first coding and were discussed by the two first authors and the corresponding author to clarify initial coding decisions by using triangulation. After a consensus was reached, a group-level analysis was conducted and the codes were combined into minor themes and then consolidated into major themes. The two first authors kept noting patterns and relationships between the categories (28), and the consensus pertaining to themes and their interpretations were discussed with all the authors.

## Results

### Demographics of Participants

A total of 23 participants participated in this study. The mean age of participants was 44.9 years, ranging from 23 to 75 years old. 13 (57.5%) were male and 10 (42.5%) were female. The mean dialysis vintage was 6.2 years. Additionally, most participants received high school education (60.8%) and had job insurance (69.6%). The monthly income of 47.8% participants were more than 2,800 RMB and the majority of participants (87%) were living with families. Table 1 shows the demographic details of the participants in the study.

**Table 1:** Demographics of participants (*n* = 23).

**Characteristics**	**Categories**	**Mean ±SD/*n* (%)**
Age		44.9 ± 14.6
Gender	Male/female	13 (56.5)/10 (43.5)
BMI		22.3 ± 4.0
Dialysis vintage (years)		6.2 ± 5.3
Residence	City/rural	16 (69.6)/ 7 (30.4)
Education level	Illiterate/Primary school/Junior high school/Senior high school/College/University	1 (4.3)/3 (13.0)/7 (30.4)/ 7 (30.4)/1 (4.3)/4 (17.4)
Medical insurance	Job insurance/Pension insurance/ Rural medical insurance/Paid by self	16 (69.6)/4 (17.4)/2 (8.7)/1 (4.3)
Income	>2,800 RMB/month/1,300-2,800RMB/month/ <1,300 RMB/month	11 (47.8)/8 (34.8)/4 (17.4)
Living with family	Yes/No	20 (87.0)/3 (13.0)

*SD, standard deviation; BMI, Body Mass Index*.

Patients' real experience, perceptions and attitudes to diet intake were explored and their dietary habits and behaviors, dietary therapy and herbal medicine, diet preferences and taboos, and dietary knowledge attainment impacted by the Chinese culture were synthesized. Table 2 presents examples of the themes in the study.

**Table 2:** Quotations about diet influenced by culture in patients undergoing HD.

**Major themes**	**Minor themes and coding**	**Exemplar quotations from patients undergoing HD**
Dietary habits influenced by culture	Neglect of sodium restriction ①nearly no one mentioned sodium restriction ②Only restrict too salty food	“About diet restriction, I know we need to control phosphorus and potassium intake...both hyperphosphatemia and hyperkalemia are very dangerous.” (man, 56 years) “I tried not to eat any pickles as they are too salty” (woman, 31 years)
	Maintaining low calcium intake ①bean products are perceived to be harmful ②not sure if vegetables are edible ③dairy products were not popular in patients	“Tofu seems harmful to us. I don't know the exact reason, but I don't eat it.” (male, 65 years) “I am not sure if I can eat dark leafy greens. It contains too much potassium, I think.” (woman, 49 years) “I am not used to drinking milk. I heard that it contains high-phosphate levels as well.” (man, 64 years)
Dietary therapy and herbal medicine	Red dates and brown sugar as excellent dietary therapy ①they are nourished ②they improve anemia	“Red dates are nourishing food. You can have several red dates and drink brown sugar to increase nutrition intake.” (woman, 49 years) “Normal hemoglobin can be maintained by eating red dates but not in excess, although I am unsure of the reason. Brown sugar can maintain normal hemoglobin levels as well” (woman, 33 years)
	Dietary herbal medicine was not accepted ①it may impair kidney ②no potential effect on promoting health	“The toxicity of herbal medicine may impair kidney function.” (male, 25 years) “I failed to see the effects of herbal medicine, you see, my disease has already progressed to hemodialysis” (man, 53 years)
Dietary preferences and taboos	Dietary preferences influenced by culture ①eggs as a preference for protein source ②broth as a preference for nutrition intake Dietary taboos influenced by culture ①seafood as a taboo due to its Fa-Wu characteristics	“I have an egg everyday, but only eat egg white.” (man, 62 years) “the hospital provides us some food during the dialysis in the afternoon. I only have an egg.” (man, 45 years) “nourishing substances are all inside the broth.”(woman, 49 years) “we all know fish or fishy-flavor food is Fa-Wu. It may deteriorate our disease. ” (woman, 65 years)
Dietary behaviors influenced by social culture	Dietary knowledge attainment impacted by culture ①deny to challenge experts ②feel ashamed of actively consulting with health care professionals No interest in dining out ①economic factor ②unhealthy diet	“Doctors and nurses know more about the disease than us, we don't have to disturb them with our questions. They will tell us about it anyway.” (man, 64 years) “They have numerous patients in the unit. It is impolite to disturb them with my questions.” (woman, 48 years) “I don't like dining out. There are too oily and too much ajinomoto in the dish. I am a patient, and the dish at the restaurant is not suitable for me” (woman, 49 years); “Eating at the restaurant is too expensive for me to afford. I have been doing hemodialysis for ten years, and the disease is a heavy economic burden for my family.” (man, 45 years)

### Dietary Habits Influenced by Culture

Nearly no participants actively mention that salt or sodium should be restricted when describing food restriction management. Only two participants stated they avoided the intake of extremely salty food in their daily lives. “I tried not to eat any pickles as they are too salty” (woman, 31 years). “I know extremely salty food makes me thirsty. But I have meals with my family, and it is very hard for them to cook based on my needs for a long time” (man, 62 years). Only one participant believed that calcium fortification was crucial for them. None of participants actively mentioned the relationship between food and calcium intake. Bean products are Chinese popular traditional food. However, nearly all patients expressed that bean products were not suitable food for them. Patients were uncertain about the reason they rejected bean products. A couple of them stated, “Tofu contains a high quantity of protein that probably cannot be eliminated via hemodialysis” (woman, 32 years). A few patients believed bean products are detrimental for controlling hyperphosphatemia, and most participants stated, “I have no idea about the reason for its restriction. It probably contains high phosphorus” (man, 77 years). Participants were confused whether vegetables were edible for patients undergoing HD due to high potassium content in them. Some patients stated, “Chinese cabbage, leek, cabbage, and spinach should be restricted for us. They contain excess potassium” (man, 62 years). However, a few participants perceived the vegetables mentioned above as permitted and even beneficial for their health. One patient stated, “Chinese cabbage and dark leafy greens are vegetables that we can eat. They are as important as meat in providing nutrition” (man, 64 years). Similarly, although only six patients expressed that they preferred to consume milk as a protein source, none of participants associated dairy with calcium source. The most prominent reasons for rejecting dairy products were “unpopular flavor” (woman, 30 years), “high-phosphate levels” (man, 64 years), and “excess fluid intake” (man, 65 years).

### Dietary Therapy and Herbal Medicine

A few participants mentioned that protein intake could lead to an increase in hematocrit or hemoglobin levels when elaborating the benefits of protein intake. Furthermore, the patients also believed that besides protein intake, it is necessary to consume brown sugar and red dates to maintain normal hemoglobin levels. One patient stated “I know that keeping myself nourished is imperative to guarantee normal hemoglobin. Normal hemoglobin can be maintained by eating red dates but not in excess, although I am unsure of the reason. Brown sugar can maintain normal hemoglobin levels as well” (woman, 33 years). None of the participants mention that herbal medicine was included in their daily diet. When participants were asked to make a detailed inquiry about herbal medicine, one participant stated, “All medicine have toxicity to some degree, herbal medicine also has side effects. We have impaired kidney function, so toxicity can't be eliminated through kidneys” (man, 46 years). Some patients stated that they had used some herbal medicine when the kidney disease was diagnosed. However, “I failed to see the effects of herbal medicine, you see, my disease has already progressed to hemodialysis” (man, 53 years).

### Dietary Preferences and Taboos

Most participants believed that consuming adequate amounts of meats, eggs and milk is an effective strategy for sufficient protein intake. However, nearly all participants reviewed eggs as the main and efficacious “protein supplement” in their daily lives. One 64-year-old male patient even stated, “An egg a day is enough for protein intake. I eat the yolk as well, and I fry eggs in excess oil.” However, most patients did not consume the egg yolk; some patients ascribed it to the yolk being rich in phosphorus, and some participants stated that it is because of its high cholesterol level. Additionally, an overwhelming majority of the participants expressed being uncritical about diet or that no ingredient was prohibited in their recipes, except for seafood. Several patients expressed that they strictly avoided consuming seafood, and the rest mentioned that they only consumed a fairly small quantity of it. Their reasons were that seafood is “Fa-Wu (the food which triggers evil and causes health problems)” (man, 62 years) and “phosphorus-rich or itch-inducing” (man, 45 years; man, 48 years). Besides it, nearly all patients expressed their concern regarding broth intake. The most prominent reasons behind it were “excess fluid intake” (woman, 33 years), “high-phosphate levels” (man, 56 years), “fat-rich” (woman, 49 years), and “high-purine levels” (man, 62 years). However, there were still a few patients who believed that the nutrients of the food had been melted inside the broth. Hence, “we should drink broth due to its nourishing substances if it is possible” (women, 58 years).

### Dietary Knowledge Attainment and Behavior Influenced by Social-Cultural Factors

The dietary knowledge source of the overwhelming majority of the participants was their experience after being diagnosed with kidney diseases, although a few participants expressed that health care professionals once provided dietary recommendations to them. Only three patients expressed that it would be possible for them to actively consult with their health care professionals about diet. The reasons they shared with us included, “Doctors and nurses know more about the disease than us, we don't have to disturb them with our questions. They will tell us about it anyway”(man, 64 years), “Doctors and nurses have numerous patients in the unit. It is impolite to disturb them with my questions” (woman, 48 years) and “They have hectic schedules to fail to provide dietary guidance” (man, 65 years). Concerning dining out, nearly no participants expressed that they were willing to eat at restaurants, and even others' homes. Most participants reported that they might occasionally eat out of home 1–2 times every month. Patients ascribed this low frequency to cost and unhealthy diet. A few participants stated, “I don't like dining out. There are too oily and too much ajinomoto in the dish. I am a patient, and the dish at the restaurant is not suitable for me” (woman, 49 years); “Eating at the restaurant is too expensive for me to afford. I have been doing hemodialysis for 10 years, and the disease is a heavy economic burden for my family” (man, 45 years). Even a patient reviewed eating out of home as wasting due to loss of appetite. He stated, “It is not economical for me to eat at a restaurant, because I can only eat a little bit of food due to my poor appetite” (woman, 33 years).

## Discussion

It has long been known that diet and nutrition are crucial to relieve systemic inflammation (29) and muscle wasting (30), improve quality of life (31), and decrease mortality risk in patients undergoing hemodialysis. A thorough understanding of diet in patients is a fundamental element in designing or conducting nutritional interventions. The evaluation system to choose lifestyle activities or not for people with chronic diseases is closely associated with their culture (32, 33). In light of the underlying relationship between diet and culture in patients undergoing maintenance hemodialysis, the qualitative study was conducted to understand Chinese patients' diet influenced by culture.

Nearly no participants actively mention the restriction of dietary salt and sodium when describing food restriction management. It potentially indicated that salt or sodium intake or restriction was not a concern for the patients in this study. The neglect of dietary salt restriciton may be ascribed to traditional Chinese diets, which are rich in salt. The mean daily salt intake of the Chinese population was 12 g in a national survey of nutrition and health status (34). Based on the data from the China National Nutrition and Health Surveillance (CNNHS) 2010–2012, the average sodium intake was 5,013 mg/day when being adjusted for energy to 2,000 kcal/day. 92.6% of adults' sodium intake exceeded the standard in the Chinese proposed intake for preventing non-communicable chronic diseases (PI-NCD) (35). This traditional diet habit may be profoundly grounded in the Chinese population, including Chinese patients undergoing hemodialysis, which was corroborated by some participants' perceptions that sodium could be restricted by only avoiding the intake of extremely salty food. Additionally, it was difficult for the Chinese patients undergoing HD to strictly restrict their salt or sodium intake. First, the group dining system has long been a tradition in Chinese. Second, caregivers in China believe that they are obligated to take good care of their sick family members by cooking for them (36). Almost none of the patients in the study needed to cook for themselves. The traditional dietary habit causes difficulties in adhering to strict salt or sodium restrictions for the whole family during their family member's long duration of hemodialysis.

Another dietary habit influenced by traditional Chinese culture is calcium intake. Chinese residence maintain low calcium intake for a long-term, which has been revealed based on nationally represented population surveys (37, 38). In western countries, calcium sources mainly include milk, cheese, legume and vegetables, in which dairy products are optimal and the predominant food rich in calcium (39). However, an investigation involving 9 provinces in China showed that vegetables (40%), bean products (20%) are main calcium sources for Chinese population (40). Although dairy products has been increased in Chinese recipe in the past decades of years, only 12% of calcium intake originates from dairy products (40). Participants in the study, like most Chinese people (41), even Chinese immigrants living in other countries (42), may have low dairy consumption. A multicenter investigation across four countries has revealed that the lower dairy intake in the Chinese participants likely led to the lower calcium intake between these countries (13). Admittedly, not everyone is fond of dairy products, and Chinese patients undergoing HD can increase the intake of calcium through consuming vegetables and bean products. However, obviously, Chinese people failed to maintain sufficient calcium intake through vegetables and bean products (40). Especially, patients undergoing HD were concerned about vegetables intake due to their potassium contents. Furthermore, patients held a belief that bean products may impair kidney function due to high-purine or high-phosphorus. Therefore, traditional low calcium intake, including low intake of dairy, vegetables and bean products may partly explain the high levels (36.0%) of hypocalcemia reported in data from Chinese patients with hemodialysis (43) except for the influence of bone mineral disease or parathyroid hormone on serum calcium levels.

There was no concept of protein in traditional Chinese culture, leading to confusion between protein and nourishing substances in participants. Chinese people believe nutritious food is multifunctional, including strengthening the immunity system and improving anemia. It may lead to the finding in the present study in which a few patients undergoing hemodialysis still misunderstood the relationship between protein intake and hemoglobin. They considered protein and other nutritious food intakes as a guaranteed method of relieving anemia. Traditional Chinese beliefs about dietary therapy were corroborated by the evidence of the patients' awareness of the increase in red dates and brown sugar intake to improve anemia. According to the Compendium of Materia Medica, dates are considered to be a Chinese traditional medicine that nourishes blood (partly by improving anemia). Zhang et al. believed that the combined effect of polysaccharides, cyclic adenosine, vitamins, inorganic salts, and other active components of dates nourishes the blood (44). Meanwhile, an increasing number of questions have been raised about the relationship between dates and anemia. The iron content of 2.4 mg in 100 g dates is much lower than that contained in animal-based foods. There is insufficient evidence of the beneficial effect of dates on iron deficiency anemia, as stated in a systematic review (45). Similarly, the Compendium of Materia Medica also indicates that brown sugar is perceived as a superfood that provides energy and nourishes the blood. To date, it is widely used by women with menstrual disorders and new mothers. A review indicated many health effects of brown sugar related to immunology, anti-toxicity, and cytoprotective ability (46). However, few publications provided sufficient documentation or evidence of improving anemia. Additionally, the iron content of approximately 2 mg in 100 g brown sugar is incapable of meeting the 20 mg/d iron requirement of healthy adult women. In terms of dietary herbal medicines, although some participants in the study once had tried, all of them expressed that they didn't consume them at that moment. Two traditional dietary medicine consisting of herbal tea and herbal soups have been widely used for several centuries in China. However, it is mainly popular in the local populations of southern China. The participants in the study come from the east of China. Potentially, the impact of traditional culture is diverse on different regions. The study identified that Chinese culture failed to significantly influence the consumption of herbal medicine in patients undergoing hemodialysis in eastern China.

The patients undergoing HD in the study perceived the intake of protein to be crucial. They reviewed meats, eggs and dairy products as protein sources and ignored protein contributed by plant products. However, increasing studies showed that plant protein are associated with favorable kidney disease outcomes, and even with low mortality in those with estimated glomerular filtration rate < 60 ml/min/1.73 m^2^ (47, 48). Additionally, participants' main source of protein in the study was egg rather than meat or other animal foods. Eggs long have been reviewed as one of the most important and nutritious food in Chinese diet culture. Its value is even utterly beyond other nourishing substances. A comparative study has shown that daily egg consumption (53.4%) was significantly higher in Chinese students compared to international students (32.8%) (49). It is also confirmed by the dietary habits of postpartum women in China. A comparative study regarding food intake during puerperium between civic and rural areas of Shandong Province found that the most popular food during puerperium were eggs in civic (96.17%) and rural (98.5%) women. Eggs were even regarded as staple food in rural postpartum women, accounting for the highest proportion (71.28%) of diet during puerperium (50). Additionally, most patients in the present study considered yolk as phosphorus-rich food. However, they still preferred egg white as the more effective strategy to promote protein intake. Restriction of the intake of the yolk is consistent that the phosphorus content in the yolk (586 mg/100 g) represents one of the highest concentrations of phosphorus in naturally occurring sources (51). Nevertheless, patients' attitudes toward egg yolk and egg white intake indicated the importance of encouraging patients to increase their protein intake sources and understand the common food sources of phosphorus, although egg white is one of the high-quality protein sources (52). The broth is a diet preference in a few of participants as well, although there were concerns due to its fluid overload, high fat and purine, and phosphorus-rich. These patients' beliefs to broth were in agreement with the Chinese proverb “Having soup is better than eating meat”, which holds that all fine nutritious substances of food are in soup. Stewing soup considered as optimal diet optimal diet has been widely consumed in the south of China. However, modern medicine and studies have confirmed that the overwhelming amount of protein remained in the food rather than in soup. Furthermore, soup, especially broth contains large amounts of purine and nitrogen substances (53) that are harmful to health or disease progression in patients undergoing hemodialysis.

Seafood was restricted by the participants partly due to its high phosphorus or itch-inducing. The impact of phosphorous contents of seafood on patients undergoing hemodialysis is still full of debate. An investigation examining the nutrients included in 14 sea fish species found that the highest phosphorus contents per 100 g of fish were 227.52 mg and the lowest was 50.86 mg (54), which remarkably lower than ones in red meat (311 mg/100 g) and egg yolk (586 mg/100 g). Increasing studies have shown seafood was potentially an excellent source of low-fat protein for patients undergoing HD (51). However, apart from phosphorus-rich of seafood, a few participants expressed concern about seafood as “Fa-Wu (stimulating food)”. Fa-Wu is the food forbidden in sick or vulnerable people. It is one of the important compositions in TCM. The Fa-Wu belief holds the traits of promoting wind (roughly refers to pernicious influence factors that can invade bodies and become a destructive influence) and provoking Qi (roughly means energy), resulting in the disorders of Qi-blood in the body or deterioration of diseases. Fish or food with fishy flavor is the most commonly seen Fa-Wu in various Chinese medical literature (55), which was corroborated by the finding of the study, indicating the strong influence of the Fa-Wu belief on patients' perceptions or behavior about diet taboos.

A low frequency of dining out was reported in most participants in the study, which might be ascribed to the influence of social culture in the Chinese population. Chinese people are family-oriented and they are more willing to cook and eat at home. This explains the reason that it is hard to accept eating at the restaurant at the Spring Festival. A qualitative study exploring diet and culture found that even Chinese American children had been limited the frequency of eating at restaurants, ranging from once a week to only on special occasions (42). A systematic review revealed that eating out of home was linked with higher socioeconomic status (52), which is consistent with the participants' concern about cost. Especially for patients undergoing hemodialysis, long-term hemodialysis has been an economic burden in China. Additionally, the review also found that eating out of home was associated with higher energy and fat intake and lower micronutrient intake (52), which is similar to the participants' concern about the influence of dining out on health. Chinese people believe that food out of home is added more oil and ingredients, which is regarded as unhealthy and even pernicious. Traditional Chinese culture holds that the kidney is an organ for eliminating ‘toxicity'. Hence, patients undergoing hemodialysis had more concern that their impaired kidney function was unable to get rid of the increased toxicity due to eating out of home. Another diet characteristic impacted by social culture was patients' social sensitivity when gaining dietary knowledge (14). It was shown that participants in the study would not actively consult with health care professionals or express their concerns. Chinese harbored a deep-seated evasive attitudes when it came to challenging experts. Addtionally, it is well known that Chinese population always hold implicit attitudes to some sensitive topics, which has been influenced by social culture for thousand of years as well.The evasive and implicit attitudes influenced by culture in the study may cause patients to fail to obtain dietary knowledge or follow the prescribed regimen, which in turn, lead to potentially suboptimal dietary intake.

## Conclusion

Diet in Chinese patients undergoing HD were strongly influenced by culture, embodied in traditional dietary habits for salt and calcium intake, dietary therapy with brown sugar and red dates, dietary preference for eggs and broth, and dietary taboos for seafood. Additionally, Chinese social culture may affect the dietary behavior regarding eating at home and dietary knowledge attainment due to the rejection of challenging experts. It is urgently required for health care providers to appreciate the influence of Chinese culture on dietary management in patients undergoing HD. While providing appropriate counseling for patients on how to improve dietary intake, doctors and nurses need to be aware of culturally sensitive dietary education or interventions. Diet management and nutritional status can be potentially enhanced by understanding Chinese culture and how they influence dietary habits and behaviors in patients undergoing HD.

## Data Availability Statement

The raw data supporting the conclusions of this article will be made available by the authors, without undue reservation.

## Ethics Statement

The studies involving human participants were reviewed and approved by Affiliated Hospital of Nantong University. The patients/participants provided their written informed consent to participate in this study.

## Author Contributions

YS and JW approached and recruited participants from hemodialysis units and conducted the interviews, independently transcribed each interview and completed the first cycle of coding. Their notes using to keep a track of rationale for coding were double-checked with the first coding and were discussed by YS, JW, and XC to clarify initial coding decisions. After a consensus was reached, further analysis including combination of the subcategories and consolidation of categories were completed by all authors in the study. Additionally, JW participated in the writing and editing English for the article. HL, XC, and MZ work at hemodialysis units and assisted the recruitment and explanation before the completion of consent forms. All authors contributed to the article and approved the submitted version.

## Funding

The study is funded by Jiangsu Students' Innovation Training Program (202010304111Y) and Nantong University Doctoral Funding (20B17).

## Conflict of Interest

The authors declare that the research was conducted in the absence of any commercial or financial relationships that could be construed as a potential conflict of interest.

## Publisher's Note

All claims expressed in this article are solely those of the authors and do not necessarily represent those of their affiliated organizations, or those of the publisher, the editors and the reviewers. Any product that may be evaluated in this article, or claim that may be made by its manufacturer, is not guaranteed or endorsed by the publisher.
