# Corrigendum: Diet and Culture Among Chinese Patients Undergoing Hemodialysis: A Qualitative Study

**DOI:** 10.3389/fnut.2022.967573

**Published:** 2022-06-29

**Authors:** Yan Song, Jing Wang, Huan Liu, Xiaolan Chen, Minqi Zhan

**Affiliations:** ^1^Department of Fundamentals of Nursing, Medical School, Nantong University, Nantong, China; ^2^Department of Medical Nursing, School of Nursing, Fudan University, Shanghai, China; ^3^Department of Nephrology, Affiliated Hospital of Nantong University, Nantong, China

**Keywords:** hemodialysis, culture, diet, nutrition, qualitative research

In the published article, there was an error in affiliation 3. Instead of “Department of Nephrology, Affiliated Hospital of Nantong, Nantong, China,” it should be “Department of Nephrology, Affiliated Hospital of Nantong University, Nantong, China.”

The authors apologize for this error and state that this does not change the scientific conclusions of the article in any way. The original article has been updated.

## Publisher's Note

All claims expressed in this article are solely those of the authors and do not necessarily represent those of their affiliated organizations, or those of the publisher, the editors and the reviewers. Any product that may be evaluated in this article, or claim that may be made by its manufacturer, is not guaranteed or endorsed by the publisher.

